# [Retracted] Downregulation of δ opioid receptor by RNA interference enhances the sensitivity of BEL/FU drug‑resistant human hepatocellular carcinoma cells to 5‑FU

**DOI:** 10.3892/mmr.2023.12974

**Published:** 2023-03-06

**Authors:** Bo Tang, Zhigao Hu, Yang Li, Shengguang Yuan, Zhenran Wang, Shuiping Yu, Songqing He

Mol Med Rep 13: 59–66, 2016; DOI: 10.3892/mmr.2015.4511

Subsequently to the publication of the above article, and a Corrigendum that was published with the intention of showing corrected data for the flow cytometric plots shown in Fig. 3 (DOI: 10.3892/mmr.2018.9415; published online on August 21, 2018), it was drawn to the Editors’ attention by a concerned reader that the β-actin agarose gel electrophoretic blots shown in Fig. 1A were strikingly similar to data appearing in different form in another article by different authors at a different research institute which had already been published elsewhere prior to this paper's submission to *Molecular Medicine Reports*. Owing to the fact that the contentious data had already been published else prior to its submission to *Molecular Medicine Reports*, the Editor has decided that this paper should be retracted from the Journal. The authors were asked for an explanation to account for these concerns, but the Editorial Office did not receive a satisfactory reply. The Editor apologizes to the readership for any inconvenience caused.

